# Longevity biotechnology: bridging AI, biomarkers, geroscience and clinical applications for healthy longevity

**DOI:** 10.18632/aging.206135

**Published:** 2024-10-16

**Authors:** Yu-Xuan Lyu, Qiang Fu, Dominika Wilczok, Kejun Ying, Aaron King, Adam Antebi, Aleksandar Vojta, Alexandra Stolzing, Alexey Moskalev, Anastasia Georgievskaya, Andrea B. Maier, Andrea Olsen, Anja Groth, Anna Katharina Simon, Anne Brunet, Aisyah Jamil, Anton Kulaga, Asif Bhatti, Benjamin Yaden, Bente Klarlund Pedersen, Björn Schumacher, Boris Djordjevic, Brian Kennedy, Chieh Chen, Christine Yuan Huang, Christoph U. Correll, Coleen T. Murphy, Collin Y. Ewald, Danica Chen, Dario Riccardo Valenzano, Dariusz Sołdacki, David Erritzoe, David Meyer, David A. Sinclair, Eduardo Nunes Chini, Emma C. Teeling, Eric Morgen, Eric Verdin, Erik Vernet, Estefano Pinilla, Evandro F. Fang, Evelyne Bischof, Evi M. Mercken, Fabian Finger, Folkert Kuipers, Frank W. Pun, Gabor Gyülveszi, Gabriele Civiletto, Garri Zmudze, Gil Blander, Harold A. Pincus, Joshua McClure, James L. Kirkland, James Peyer, Jamie N. Justice, Jan Vijg, Jennifer R. Gruhn, Jerry McLaughlin, Joan Mannick, João Passos, Joseph A. Baur, Joe Betts-LaCroix, John M. Sedivy, John R. Speakman, Jordan Shlain, Julia von Maltzahn, Katrin I. Andreasson, Kelsey Moody, Konstantinos Palikaras, Kristen Fortney, Laura J. Niedernhofer, Lene Juel Rasmussen, Liesbeth M. Veenhoff, Lisa Melton, Luigi Ferrucci, Marco Quarta, Maria Koval, Maria Marinova, Mark Hamalainen, Maximilian Unfried, Michael S. Ringel, Milos Filipovic, Mourad Topors, Natalia Mitin, Nawal Roy, Nika Pintar, Nir Barzilai, Paolo Binetti, Parminder Singh, Paul Kohlhaas, Paul D. Robbins, Paul Rubin, Peter O. Fedichev, Petrina Kamya, Pura Muñoz-Canoves, Rafael de Cabo, Richard G. A. Faragher, Rob Konrad, Roberto Ripa, Robin Mansukhani, Sabrina Büttner, Sara A. Wickström, Sebastian Brunemeier, Sergey Jakimov, Shan Luo, Sharon Rosenzweig-Lipson, Shih-Yin Tsai, Stefanie Dimmeler, Thomas A. Rando, Tim R. Peterson, Tina Woods, Tony Wyss-Coray, Toren Finkel, Tzipora Strauss, Vadim N. Gladyshev, Valter D. Longo, Varun B. Dwaraka, Vera Gorbunova, Victoria A. Acosta-Rodríguez, Vincenzo Sorrentino, Vittorio Sebastiano, Wenbin Li, Yousin Suh, Alex Zhavoronkov, Morten Scheibye-Knudsen, Daniela Bakula

**Affiliations:** 1 Institute of Advanced Biotechnology and School of Medicine, Southern University of Science and Technology, Shenzhen, China; 2 Max Planck Institute for Biology of Ageing, Cologne, Germany; 3 Institute of Aging Medicine, College of Pharmacy, Binzhou Medical University, Yantai, China; 4 Anti-aging Innovation Center, Subei Research Institute at Shanghai Jiaotong University, China; 5 Shandong Cellogene Pharmaceutics Co. LTD, Yantai, China; 6 Duke Kunshan University, Kunshan, Jiangsu, China; 7 Division of Genetics, Department of Medicine, Brigham and Women's Hospital, Harvard Medical School, Boston, MA 02108, USA; 8 Foresight Institute, San Francisco, CA 91125, USA; 9 Excellence Cluster on Cellular Stress Responses in Aging-Associated Diseases (CECAD), University of Cologne, Cologne, Germany; 10 Department of Biology, Division of Molecular Biology, Faculty of Science, University of Zagreb, Zagreb, Croatia; 11 Centre for Biological Engineering, Wolfson School of Mechanical, Electrical and Manufacturing Engineering, Loughborough University, Loughborough, UK; 12 Institute of Biogerontology, Lobachevsky University, Nizhny Novgorod, Russia; 13 HautAI OÜ, Tallinn, Estonia; 14 Healthy Longevity Translational Research Program, Yong Loo Lin School of Medicine, National University of Singapore, Singapore; 15 California Institute of Technology, Pasadena, CA 91125, USA; 16 Novo Nordisk Foundation Center for Protein Research (CPR), Faculty of Health and Medical Sciences, University of Copenhagen, Copenhagen, Denmark; 17 Max Delbrück Center for Molecular Medicine, Berlin, Germany; 18 The Kennedy Institute of Rheumatology, Oxford, UK; 19 Department of Genetics, Stanford University, Stanford, CA 94305, USA; 20 Insilico Medicine AI Limited, Level 6, Masdar City, Abu Dhabi, UAE; 21 Systems Biology of Aging Group, Institute of Biochemistry of the Romanian Academy, Bucharest, Romania; 22 America's Frontier Fund, VA 23320, USA; 23 Department of Biology, School of Science, Center for Developmental and Regenerative Biology, Indiana University - Purdue University Indianapolis, Indianapolis Indiana 46077, USA; 24 Centre for Physical Activity Research, Rigshospitalet and University of Copenhagen, Denmark; 25 Institute for Genome Stability in Aging and Disease, CECAD Research Center, University and University Hospital of Cologne, Cologne 50931, Germany; 26 199 Biotechnologies Ltd., London, UK; 27 University College London, London, UK; 28 Molecular, Cellular, And Integrative Physiology Interdepartmental Program, University of California Los Angeles, Los Angeles, CA 90095, USA; 29 Department of Molecular and Medical Pharmacology, David Geffen School of Medicine, University of California Los Angeles, Los Angeles, CA 90095, USA; 30 Hong Kong Quantum AI Lab, Hong Kong; 31 Zucker School of Medicine at Hofstra/Northwell, NY 10001, USA; 32 Charité - University Medicine, Berlin, Germany; 33 Lewis Sigler Institute for Integrative Genomics, Princeton University, Princeton, NJ 08540, USA; 34 Department of Molecular Biology, Princeton University, Princeton, NJ 08540, USA; 35 Laboratory of Extracellular Matrix Regeneration, Institute of Translational Medicine, Department of Health Sciences and Technology, ETH Zürich, Schwerzenbach CH-8603, Switzerland; 36 Department of Nutritional Sciences and Toxicology, University of California, Berkeley, Berkeley, CA 94720, USA; 37 Metabolic Biology Graduate Program, University of California, Berkeley, Berkeley, CA 94720, USA; 38 Endocrinology Graduate Program, University of California, Berkeley, Berkeley, CA 94720, USA; 39 Leibniz Institute on Aging, Fritz Lipmann Institute, Friedrich Schiller University, Jena, Germany; 40 Longevity Center Europe, Warsaw, Poland; 41 Centre for Psychedelic Research, Dpt Brain Sciences, Imperial College London, UK; 42 Blavatnik Institute, Department of Genetics, Paul F. Glenn Center for Biology of Aging Research at Harvard Medical School, Boston, MA 02108, USA; 43 Signal Transduction and Molecular Nutrition Laboratory, Kogod Aging Center, Department of Anesthesiology and Perioperative Medicine, Mayo Clinic College of Medicine, Rochester, MN 55902, USA; 44 School of Biology and Environmental Science, Belfield, Univeristy College Dublin, Dublin 4, Ireland; 45 BioAge Labs, Richmond, CA 94530, USA; 46 Buck Institute for Research on Aging, Novato, CA 94945, USA; 47 Research and Early Development, Maaleov 2760, Denmark; 48 VitaDAO, Ottawa, ON K1V 9K6, Canada; 49 Department of Clinical Molecular Biology, University of Oslo and Akershus University Hospital, Lørenskog, Norway; 50 Department of Medical Oncology, Renji Hospital, School of Medicine, Shanghai Jiao Tong University, Shanghai, China; 51 Novo Nordisk Foundation Center for Protein Research, University of Copenhagen, Copenhagen 2200, Denmark; 52 Novo Nordisk Foundation Center for Basic Metabolic Research, University of Copenhagen, Copenhagen N 2200, Denmark; 53 European Research Institute for the Biology of Ageing (ERIBA), University of Groningen, University Medical Center Groningen, Groningen, The Netherlands; 54 Insilico Medicine Hong Kong Ltd., Hong Kong Science and Technology Park, Hong Kong SAR, China; 55 Rejuveron Life Sciences AG, 8952 Schlieren, Switzerland; 56 Health, Nutrition and Care, DSM-Firmenich, Kaiseraugst, Switzerland; 57 LongeVC, Riga, Latvia; 58 InsideTracker, Cambridge, MA 02114, USA; 59 Department of Psychiatry, Columbia University, New York, NY 10012, USA; 60 Maxwell Biosciences, Austin, TX 73301, USA; 61 Division of General Internal Medicine, Department of Medicine, Mayo Clinic, Rochester, MN 55905, USA; 62 Cambrian Bio, Inc., New York, NY 10012, USA; 63 XPRIZE Foundation, Culver City, CA 90230, USA; 64 Department of Genetics Albert Einstein College of Medicine, New York, NY 10461, USA; 65 Department of Cellular and Molecular Medicine, Faculty of Health and Medical Sciences, University of Copenhagen, Copenhagen, Denmark; 66 Life Biosciences, Boston, MA 02116, USA; 67 Tornado Therapeutics, Cambrian Bio Inc. PipeCo, New York, NY 10012, USA; 68 Department of Physiology and Biomedical Engineering and Robert and Arlene Kogod Center on Aging, Mayo Clinic, Rochester, MN 55905, USA; 69 Department of Physiology and Institute for Diabetes, Obesity, and Metabolism, Perelman School of Medicine, University of Pennsylvania, Philadelphia, PA 19019, USA; 70 Retro Biosciences, Inc., Redwood City, CA 94061, USA; 71 Center on the Biology of Aging, Department of Molecular Biology, Cell Biology and Biochemistry, Brown University, Providence, RI 02860, USA; 72 Shenzhen Key Laboratory of Metabolic Health, Center for Energy Metabolism and Reproduction, Shenzhen Institute of Advanced Technology, Chinese Academy of Sciences, Shenzhen, China; 73 Private Medical, San Francisco, CA 94107, USA; 74 Faculty of Health Sciences Brandenburg and Faculty of Environment and Natural Sciences, Brandenburg University of Technology Cottbus-Senftenberg, Senftenberg 01968, Germany; 75 Department of Neurology and Neurological Sciences, Stanford University School of Medicine, Stanford, CA 94305, USA; 76 Ichor Life Sciences, Inc., LaFayette, NY 13084, USA; 77 Department of Physiology, Medical School, National and Kapodistrian University of Athens, Athens, Greece; 78 Institute on the Biology of Aging and Metabolism, Department of Biochemistry, Molecular Biology and Biophysics, University of Minnesota, Minneapolis, MN 55414, USA; 79 Center for Healthy Aging, Department of Cellular and Molecular Medicine, University of Copenhagen, Denmark; 80 Nature Biotechnology, Springer Nature, London, UK; 81 Longitudinal Studies Section, Translational Gerontology Branch, National Institute on Aging, Baltimore, MD 21201, USA; 82 Rubedo Life Sciences, Sunnyvale, CA 94043, USA; 83 Turn Biotechnologies, Mountain View 94039, CA, USA; 84 Phaedon Institute, Oakland, CA 94501, USA; 85 Institute of Biochemistry of the Romanian Academy, Romania; 86 Fertility and Research Centre, Discipline of Women's Health, School of Clinical Medicine, University of New South Wales, Sydney, New South Wales, Australia; 87 Longevity Biotech Fellowship, Longevity Acceleration Fund, Vitalism, SF Bay, CA 94101, USA; 88 Department of Biochemistry, Yong Loo Lin School of Medicine, National University of Singapore, Singapore 117608, Singapore; 89 Boston Consulting Group, Boston, MA 02210, USA; 90 Leibniz-Institut Für Analytische Wissenschaften-ISAS-E.V., Dortmund, Germany; 91 Repair Biotechnologies, Inc., Syracuse, NY 13210, USA; 92 Sapere Bio, Triangle Research Park, NC 27213, USA; 93 Holmusk, New York, NY 10012, USA; 94 AniBiome, Zagreb, Croatia; 95 Institute for Aging Research, Albert Einstein College of Medicine, Bronx, NY 10452, USA; 96 Molecule, Neuhausen am Rheinfall, Switzerland; 97 Institute on the Biology of Aging and Metabolism and the Department of Biochemistry, Molecular Biology, and Biochemistry, University of Minnesota, Minneapolis, MN 55111, USA; 98 Gero PTE, Singapore, Singapore; 99 Insilico Medicine Canada Inc., Montreal, Quebec H3B 4W8 Canada; 100 Altos Labs Inc., San Diego Institute of Science, San Diego, CA 92121, USA; 101 Translational Gerontology Branch, Intramural Research Program, National Institute on Aging (NIH), Baltimore, Maryland 21201, USA; 102 Huxley Building, School of Applied Sciences, University of Brighton, Brighton, UK; 103 Biolytica, Zug, Switzerland; 104 Deciduous Therapeutics, San Francisco, CA 94107, USA; 105 Department of Molecular Biosciences, The Wenner-Gren Institute, Stockholm University, Stockholm 10691, Sweden; 106 Department of Cell and Tissue Dynamics, Max Planck Institute for Molecular Biomedicine, Münster, Germany; 107 Healthspan Capital, El Cerrito, CA 94530, USA; 108 School of Public Health, Li Ka Shing Faculty of Medicine, The University of Hong Kong, Hong Kong; 109 Department of Physiology, Healthy Longevity Translational Research Program, Yong Loo Lin School of Medicine, National University of Singapore, Singapore; 110 Institute of Cardiovascular Regeneration, Center of Molecular Medicine, Goethe University Frankfurt, Germany; 111 Eli and Edythe Broad Center of Regenerative Medicine and Stem Cell Research, University of California Los Angeles, Los Angeles, CA 90095, USA; 112 Bioio, Inc., St. Louis, MO 63110, USA; 113 Collider Heath, London, UK; 114 Healthy Longevity Champion, National Innovation Centre for Ageing, UK; 115 Aging Institute, University of Pittsburgh School of Medicine, Pittsburgh, PA 15106, USA; 116 Sheba Longevity Center, Sheba Medical Center, Tel Hashomer, Israel; 117 Tel Aviv Faculty of Medicine, Tel Aviv University, Tel Aviv, Israel; 118 Longevity Institute, Davis School of Gerontology and Department of Biological Sciences, University of Southern California, Los Angeles, CA 90001, USA; 119 TruDiagnostic, Lexington, KY 40503, USA; 120 Department of Biology and Medicine, University of Rochester, Rochester, NY 14627, USA; 121 Department of Neuroscience, Peter O’Donnell Jr. Brain Institute, University of Texas Southwestern Medical Center, Dallas, TX 75390, USA; 122 Department of Obstetrics and Gynecology, School of Medicine, Stanford University, Stanford, CA 94301, USA; 123 Department of Neuro-Oncology, Beijing Tiantan Hospital, Capital Medical University, Beijing 100070, China; 124 Department of Obstetrics and Gynecology, Columbia University, New York City, NY 10032, USA; 125 Duke University, Durham, NC, USA; 126 Institute for Biostatistics and Informatics in Medicine and Ageing Research, Rostock University Medical Center, Rostock, Germany

**Keywords:** biotechnology, artificial intelligence, healthy longevity

## Abstract

The recent unprecedented progress in ageing research and drug discovery brings together fundamental research and clinical applications to advance the goal of promoting healthy longevity in the human population. We, from the gathering at the Aging Research and Drug Discovery Meeting in 2023, summarised the latest developments in healthspan biotechnology, with a particular emphasis on artificial intelligence (AI), biomarkers and clocks, geroscience, and clinical trials and interventions for healthy longevity. Moreover, we provide an overview of academic research and the biotech industry focused on targeting ageing as the root of age-related diseases to combat multimorbidity and extend healthspan. We propose that the integration of generative AI, cutting-edge biological technology, and longevity medicine is essential for extending the productive and healthy human lifespan.

## INTRODUCTION

**Figure 1:**
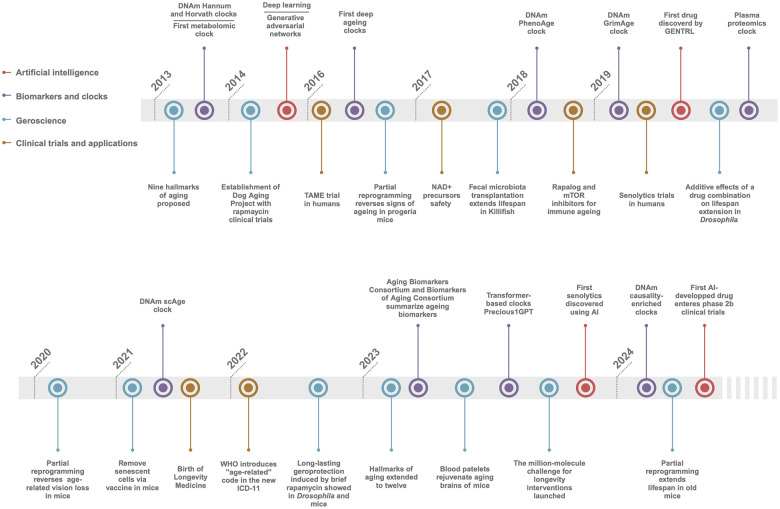
**Timeline of longevity biotechnology.** Key breakthroughs the AI, Biomarkers and clocks, Geroscience, and Clinical trials and applications in ageing and longevity fields since 2013.

The landscape of ageing research and interventions targeting age-related diseases has undergone significant advancement in the past decade [1, 2]. Since the first Aging Research and Drug Discovery (ARDD) meeting in 2013, the field has evolved into a multidisciplinary arena, attracting substantial funding, spawning numerous startups, and yielding groundbreaking discoveries. It now encompasses contributions from a broad spectrum of professionals, including biologists, physicians, data scientists, and entrepreneurs, bridging the public and private sectors. This dynamic progression has been greatly accelerated by the advent of cutting-edge technologies, such as artificial intelligence (AI), comprehensive omics analyses, and innovative ageing clocks (Figure 1). These tools have catalysed advancements in our understanding of ageing processes and the development of safe and effective interventions to combat ageing and chronic illness, leading to healthspan extension.

This evolving paradigm is characterized by the synergistic integration of AI and big data analytics, which have emerged as transformative forces in the identification, characterization, and predictive analysis of ageing biomarkers [3]. AI-driven biomarker discovery is increasingly recognized as a cornerstone for advancing personalized medicine and improving healthcare outcomes. Yet, the journey from biomarker discovery to clinical translation encompasses a myriad of challenges, including rigorous validation processes and the harmonization of regulatory standards [4–6]. Overcoming these obstacles necessitates continued investment, collaboration, and innovation, underscoring the pivotal role of biomarkers in the nexus of ageing research, drug discovery, and clinical applications.

Within this context, longevity biotechnology emerges as an interdisciplinary bridge that connects AI, biomarkers discovery and deployment, geroscience, and clinical applications, aiming to redefine healthcare paradigms towards achieving healthy longevity. This review, compiled by the ARDD speakers following the 2023 ARDD event, aims to encapsulate the latest strides in longevity biotechnology. It highlights the convergence of AI-driven approaches with traditional research methodologies, underscoring the potential of this union in addressing the complex challenges posed by ageing and age-related diseases. By delving into AI-empowered biomarker development, mechanisms of ageing, gerotherapeutics, and the landscape of clinical trials and interventions, we endeavour to provide a comprehensive perspective on state of the art in longevity science and its implications for future healthcare strategies.

## AI in ageing research

The emergence of longevity biotechnology as a standalone industry has led to the acceleration of the convergence of AI and ageing research [3], which led to notable advancements in longevity science, with AI playing an increasing role in biomarker identification, drug discovery, and clinical practice. The link between longevity and AI is expected to become even stronger, as AI is helping to understand the wider determinants of health, including how the environment influences gene expression, yielding new insights on how to increase the human healthy lifespan [7].

### AI-driven biomarker identification

The fusion of AI with biomarker research has markedly revolutionized the way biomarkers are identified and validated in the field of ageing. Machine learning algorithms, deep learning methods, and big data analytics have facilitated the discovery of novel biomarkers of ageing crucial for disease diagnosis, prognosis, and predicting treatment outcomes [8, 9]. For example, deep learning algorithms applied to cellular images across multiple tissues identified nuclear morphology as a new universal senescence marker [10]. Yet, it is often challenging to use singular indicators as ageing biomarkers. Using panels or complex biomarkers that combine data from multiple ‘omic’ technologies is recommended [11]. It is especially important to use explainable AI to create models that can predict chronological age from non-invasive measurements. In the future, the integration of AI with emerging technologies, such as single-cell sequencing and spatial transcriptomics for the biomarker discovery for complex diseases with multifactorial etiology will be at the forefront of ageing research. Notably, it is becoming increasingly important to identify not only biomarkers that predict the onset of age-related disorders and diseases, but also therapeutic biomarkers that change in response to gerotherapeutic interventions that delay, prevent, alleviate, or treat age-related disorders and diseases and that may extend healthspan [5, 12].

### AI in drug discovery and longevity science

One of the most prominent applications of AI in the ageing field is drug discovery, where AI techniques are used to identify and design new compounds [13, 14]. Pharmaceuticals have been identified that target some of the hallmarks of ageing, which could be utilized to address age-related multimorbidities. For example, several recent findings showed machine learning or deep neural networks are able to assist in the discovery of senolytic compounds in preclinical models [15, 16], first-in-class preclinical drug candidates against Huntington’s disease [17], and novel mTOR inhibitors and repurposing hypertension drug rilmenidine for extending *C.elegans* lifespan [18, 19]. This approach has significantly accelerated the process of finding anti-ageing therapeutic solutions.

The usage of AI extends beyond drug discovery. Machine learning techniques have been employed to decode the genetic and epigenetic factors associated with longevity, such as those related to physical fitness and specific genes like ACTN3 [20]. This provides a crucial example of how AI, particularly deep learning and machine learning, accelerate longevity science by uncovering novel biomarkers and elucidating the complex genetic and epigenetic underpinnings of ageing. Furthermore, the application of hybrid quantum-classical machine learning techniques reveals new perspectives on biological age and its determinants and opens new avenues for the development of gerontechnology [21].

## Biomarkers and ageing clocks

Biomarkers and clocks play a crucial role in understanding age and age-related disease progression, predicting outcomes, and optimizing treatment strategies. Recent contributions from the Aging Biomarkers Consortium and the Biomarkers of Aging Consortium provided a comprehensive summary of the current state of biomarkers across cellular, organ, and organismal levels of ageing. These groups have also proposed a refined framework for the terminology and characterization of ageing biomarkers, offering valuable insights and data for the field [5, 12]. Furthermore, updates on the hallmarks of ageing delivered extensive information and complex biological datasets, underscoring the necessity of integrating AI with traditional biomarker discovery approaches to derive meaningful insights [1].

### Biomarker validation and clinical translation

The biomarkers of ageing need to be widely validated before being incorporated into clinical practice [4]. The ageing process is highly variable across tissues and organs [22, 23]. Moreover, the ageing process varies significantly among individuals, with stochastic variation alone can explain a significant portion of ageing dynamics. The accuracy of predictive biomarkers may plateau as stochasticity increases [24], highlighting the variability in biological ageing among individuals and the value of measuring this difference. Validation is a multistep process that defines the characteristics of biomarkers, including their reliability, accuracy, and ability to predict relevant outcomes [4, 25]. This process requires expertise in various areas, such as the biological mechanisms of ageing, the design and construction of composite biomarkers, and the validation of biomarkers across diverse population samples. Collaboration between basic scientists and clinical investigators is essential for successfully navigating this process.

Initiatives such as Biolearn, methylCIPHER, Estimage, and Clockbase have emerged to facilitate the development, validation, and comparison of ageing biomarkers [26–29]. These platforms provide standardized datasets and evaluation metrics, enabling researchers to benchmark their biomarkers against existing ones and identify areas for improvement. Such collaborative efforts are crucial for accelerating the translation of ageing biomarkers into clinical practice. To enhance rigor in the validation process, guidelines for standardization and harmonization of biomarkers across populations with unique characteristics are needed [4]. Recommendations on metrics for reporting predictive performance should also be established. Systematic validation can accelerate the clinical translation of ageing biomarkers and their use in gerotherapeutic clinical trials.

### Ageing clocks and AI

Ageing clocks are a specific type of biomarker designed to predict biological age, typically through computational models. These clocks often use a combination of biomarkers, integrating them through machine learning techniques to estimate the overall ageing rate and biological age of an individual. It has been a decade since the first-generation DNA methylation clocks were created [30]. Since then, many biological clocks have been developed, from clocks based on molecular signatures such as DNA methylation, transcriptome, lipids and glycans to physiological clocks using facial, fundus, and tongue images [31–35].

One of the limitations of most existing clocks is that often reflect changes in blood cell populations rather than intrinsic ageing. Removing CpG sites linked to T cell differentiation could be a way to measure intrinsic ageing more accurately [36]. Another limitation of most existing clocks is their lack of information on causality, which means that while ageing clocks can show correlations with ageing, they are not able to pinpoint which biological changes are causing ageing. DamAge and AdaptAge-clocks are novel epigenetic clocks that are based on causal analysis [37]. These clocks might prove to be more useful for tracking short-term interventions making them suitable and applicable for assessing nuanced effects on geroprotective and rejuvenative interventions.

Beyond the DNA methylation clocks, blood-measurement-based biological age predictors have been developed since 2016, when a deep neural network (DNN) approach was introduced to predict age from blood biochemistry data [3, 38]. Since then, several studies have built upon this concept, refining and expanding the use of blood parameters to predict biological age and health outcomes. For example, the “block clock” employs an array of blood parameters, such as complete blood count (CBC), and other parameters measured with a scale and glucose meter, demonstrating its power to forecast health and survival outcomes in mice [39]. Similarly, a new OMICmAge integrates multi-omics data, including proteomics, metabolomics, clinical information, and DNA methylation, resulting in composite metric, shows promising associations with disease, improved hazard ratios, and higher accuracy in predicting 5-year and 10-year survival rates compared to single-omic clocks [40]. However, despite the great potential of biological clocks supported by AI, the lack of uniform data exchange protocols between laboratories and units conducting development research makes it difficult to create a reliable clinical tool. Many solutions are still based on reading text documents by an AI algorithm, which carries the risk of incorrect data in the analysis.

In sum, there is an increasing demand in the field for the development of more sophisticated ageing biomarkers and clocks. These should not only predict ageing and associated risks but also elucidate the underlying mechanisms and causal relationships. Furthermore, employing AI to integrate multi-omics data for advanced ageing clocks presents a promising strategy to advance ageing research and improve healthcare outcomes.

## Mechanisms of ageing, drug discovery and gerotechnology

In recent years, our understanding of the mechanisms of ageing has significantly advanced. Of note, the proposition of twelve hallmarks of ageing includes primary hallmarks which reflect on the molecular-level damages; antagonistic hallmarks which represent the organelle and cellular response to the damage, and integrative hallmarks which reflect alternations at systemic levels [1]. The shift from the pursuit of single unified theories of ageing towards explaining ageing as a complex combination of molecular events and cellular responses has been useful towards the development of novel interventions and treatments targeting age-related diseases.

### Primary hallmarks of ageing and potential applications

The primary hallmarks of ageing include genome instability (DNA damage and repair, and genetic mutations), telomere damage and attrition, epigenetic changes to the genetic code and histones that regulate DNA accessibility, and autophagy/proteostasis that ameliorate damage to proteins and organelles [1]. These hallmarks comprise the consequences of normal metabolism and energetics and how well cells respond to that unavoidable stress. The primary hallmarks are dictated by both genetic and environmental factors and thereby are arguably the most heterogeneous and unavoidable consequences of ageing.

#### Genome integrity

Recent advancements have further delineated the mechanisms associated with the hallmarks of ageing. Among the primary hallmarks, genome instability, predominantly induced by endogenous DNA damage driven by normal metabolism, the accumulation of genetic mutations and the activity of transposable elements, along with telomeric DNA damage and attrition, the latter being the progressive shortening of telomeres at the ends of chromosomes during cell division, plays a key role in the progression of ageing and its associated diseases [1, 41]. Studies using progeroid mouse models, such as Ercc1 mutant, a model of a human progeroid syndrome, have illustrated how particular gene mutations in DNA repair genes can accelerate ageing processes, initiate most of the other hallmarks of ageing, and compromise various organ systems, illustrating the interconnectedness of hallmarks of ageing [42]. The recent discovery of the DREAM complex as a master regulator of DNA repair gene expression provides a potential pharmacological target for boosting genome integrity thus alleviating a causal mechanism that drives the ageing process [43].

#### Epigenetic alternations

The alteration of epigenetic information with age represents another pivotal mechanism influencing ageing [1]. Innovations in partial reprogramming, using Yamanaka factors and other agents, suggest that the reversal of the epigenetic landscape to a more youthful state may counteract ageing effects [44–48]. This hypothesis is supported by findings that proper maintenance of epigenetic marks is essential for preserving cell identity and mitigating ageing and cancer-related changes in the epigenome [49, 50]. Furthermore, recent research demonstrates that age-associated decline in heterochromatin levels contributes to genomic instability and diminished regenerative capacity, suggesting that interventions aimed at rejuvenating aged cells could be beneficial [51].

Given the therapeutic potential of partial reprogramming, it emerges as a highly promising strategy for combating the effects of ageing and its related diseases. Research targeting specific cell types, such as retinal ganglion cells with reprogramming techniques, reveals the potential to restore cellular function lost with age [52]. Furthermore, the development and application of mRNA-based reprogramming for skin rejuvenation exemplifies the innovative approaches being pursued to reverse age-related cellular changes, offering the non-invasive and transformative potential for anti-ageing interventions [53]. However, safety concerns of partial reprogramming, such as how to protect cell type identity during the reprogramming process or predisposition to cancer may hinder its therapeutic applications [54].

#### Autophagy and proteostasis

Macroautophagy (we here refer to it as “autophagy”) is a critical cellular process involving the turnover and recycling of organelles and proteins [55]. It plays a pivotal role in maintaining cellular homeostasis and combating the deleterious effects of protein and organelle damage that occurs during ageing. Stimulation of autophagy is sufficient to decelerate age-related pathology and extend lifespan in diverse animal models [1, 56]. Of note, a brief treatment of rapamycin in early adulthood induces sustained autophagy activation and thereby attenuates age-related gut pathology and systemic inflammation markers in mice, shedding light for further testing in clinical trials [57]. Moreover, the integration of AI has yielded promising drug candidates targeting specific autophagic processes, such as mitochondrial autophagy (mitophagy), showcasing positive outcomes in Alzheimer’s disease preclinical models [58].

Parallel to autophagy, proteostasis declines with age, contributing to a range of degenerative diseases including Alzheimer’s [59]. Intervention strategies that modulate protein synthesis, bolster chaperones like heat shock proteins (HSPs), and enhance proteasomal activities decrease toxic protein aggregates and alleviate symptoms of degenerative conditions, illustrating the critical role of proteostasis in maintaining cellular integrity and function [59]. These insights into autophagy and proteostasis deepen our understanding of cellular mechanisms underlying ageing and pave the way for novel therapeutic approaches to combat age-related diseases.

### Antagonistic hallmarks and pathways to clinical translation

Antagonistic hallmarks of ageing, including deregulated nutrient sensing, mitochondrial dysfunction, and cellular senescence, play pivotal roles in the ageing process and the development of age-related diseases [1]. These mechanisms comprise the organelle and cellular responses to the primary hallmarks of ageing and stress, and are central to the current research and clinical studies in geroscience, highlighting their significance and potential translatability into therapeutic interventions [60–62].

#### Nutrient sensing

Research into the deregulation of nutrient-sensing pathways, such as insulin/IGF-1, mTOR, and AMPK, reveals a profound impact on the ageing process and associated diseases. These pathways, when disrupted, contribute significantly to the ageing phenotype, presenting targeted opportunities for intervention. Interventions in this area focus on modulating the activity of these pathways to decelerate ageing and mitigate age-related conditions [63–65]. For example, subtly adjusting mTOR activity maintains muscle strength without promoting excessive growth, indicating a delicate balance between growth and ageing. Similarly, modulating the activity of the AMPK complex may foster metabolic health and support healthy ageing [66].

Nutrient-sensing pathways are well connected with other hallmarks of ageing. Dietary interventions like intermittent fasting and Fasting Mimicking Diet (FMD) have been explored for their potential to slow down the ageing process and prevent the onset of age-related diseases [67]. Notably, circadian alignment can enhance the effect of calorie restriction on lifespan extension in male mice, underscoring the importance of considering timing when optimizing dosing regimens of geroprotectors [68]. Furthermore, pharmacological reagents like metformin, and rapamycin, alongside natural compounds, such as spermidine, alpha-ketobutyrate, alpha-ketoglutarate and taurine, or their synergistic combinations, have been identified for targeting these nutrient-sensing mechanisms to mediate multiple hallmarks of ageing [64, 69–75]. Such interventions have the potential to alter multiple age-related hallmarks, ultimately contributing to the extension of healthy lifespan in preclinical models and potentially in humans as well.

#### Mitochondrial dysfunction

Mitochondrial dysfunction, which is closely associated with the deregulation of nutrient-sensing pathways, is increasingly recognized as a cornerstone of ageing and its associated illness. A decline in mitochondrial function and a concomitant decrease in NAD+ levels are closely linked with ageing process [76]. Reducing mitochondrial protein folding stress in the dentate gyrus improves neurogenesis and cognitive function in old mice [77]. Therapeutic approaches targeting mitochondrial dysfunction aim to restore or enhance mitochondrial health and energetic efficiency. Research findings underline the significance of CD38 in regulating NAD levels and the role of NAD levels in stem cell fate, suggesting their beneficial impact during ageing processes [78]. Trigonelline is a natural, bioactive pyridine alkaloid that is a precursor of NAD+ and restores mitochondrial function muscle in the muscle of *C.elegans* and mice [79]. Research suggests that deficiency in cardiolipin, a phospholipid which is exclusively located in mitochondria, increases fatty acid oxidation in glycolytic muscle and accelerates muscle ageing. Furthermore, interventions targeting the mitochondrial matrix, such as the inhibition of manganese-induced coenzyme Q, which regulates ATP and ROS production, hold promise [80]. These findings underscore the importance of maintaining mitochondrial function and integrity for healthy ageing and disease prevention and indicate that natural compound supplementation can be a potential strategy for slowing down muscle ageing.

#### Cellular senescence

Senescent cells plays a significant role in the physiological decline associated with ageing, making them pivotal targets for anti-ageing interventions [1, 81]. Current research efforts focus on understanding the triggers and consequences of cellular senescence, pinpointing the location of these cells and the communication with neighbouring cells, and uncovering the connection with other hallmarks of ageing [82, 83]. This comprehensive understanding aims to provide foundational knowledge for the development of therapeutics that help alleviate age-related changes that drive vulnerability to chronic diseases.

The therapeutic landscape in this domain is notably enriched by the development of senolytics and senomorphics, which are meticulously crafted to selectively eliminate senescent cells or suppress their deleterious pro-inflammatory secretions, respectively. These innovative approaches have demonstrated promise in mitigating age-associated pathologies and promoting healthspan [83]. Currently, senotherapeutics include repurposing established pharmaceuticals (many from oncology), such as dasatinib and quercetin, delving into natural compounds like fisetin, and pioneering new treatments involving extracellular vesicles (EVs), vaccines, and CAR-T therapies [83–86]. It is possible to rejuvenate senescent cells using partial reprogramming techniques, so the senescence state can be reversed [87]. These diverse approaches enhance the likelihood of tackling cellular senescence safely and effectively. Moreover, the “hit-and-run” approach to targeting senescent cells selectively aligns well with the current timelines and regulatory standards of clinical trials, providing an innovative bridge between the fields of oncology and longevity medicine [83].

In sum, the categorization of antagonistic hallmarks into deregulated nutrient sensing, mitochondrial dysfunction, and cellular senescence provides a structured framework for understanding and targeting the ageing process. Research findings in each category inform the development of targeted interventions, illustrating the promising trajectory from scientific inquiry to clinical application in the field of geroscience. Notably, the majority of clinical trials testing gerotherapeutic interventions currently underway target the antagonistic hallmarks of ageing rather than the upstream primary hallmarks of ageing or the downstream integrative hallmarks [61].

### Integrative hallmarks of ageing and therapeutic strategies

#### Chronic inflammation

Chronic inflammation and inflammaging are closely associated with functional decline and systemic ageing, yet their exact underlying sources and consequences remain to be fully elucidated [1, 88]. Notably, inflammation is often linked with other ageing hallmarks [1]. For example, recent research reveals that ageing leads to a decline in glycolysis and mitochondrial oxidative phosphorylation in myeloid cells, undermining immune cell functions and exacerbating inflammation [89]. Moreover, the response to infection is negatively impacted by the presence of senescent cells, which in turn promote further senescence when infected by viruses. Interestingly, long-lived species such as bats demonstrate remarkable immune responses to infections and viral containment, serving as an extraordinary model for unravelling the complexities of inflammation in the ageing process [90, 91].

Given the intricate relationship between chronic inflammation, age-related immune dysfunction (inflammaging) and immune-related diseases, the development of targeted interventions and treatments to mediate immune function is appealing. Novel strategies, such as inhibitors of key inflammation mediators like NLRP3, peptide-based therapeutics inspired by natural antimicrobial peptides, and immunotherapies for removing senescent or exhausted immune cells and enhancing immune function in ageing are under exploration [92]. AI-powered diagnostics and therapeutic solutions aimed at reversing gut inflammation and promoting gut health in ageing are also being developed [93]. To assess immune function, the immune longevity score (ILS) has been developed as a metric for evaluating the extensive functionality of the immune system, potentially serving as a novel ageing clock.

#### Microbiome, intercellular communication and stem cell activity

The gut microbiome has emerged as a critical factor influencing host metabolism, behaviour, and ageing [94]. Fecal microbiota transplantation (FMT) from young to old animals is sufficient to extend lifespan in killifish and reverse age-related differences in both peripheral and brain immunity in mice, highlighting the significant role of the microbiome in mediating host health and ageing [95, 96]. Healthy gut microbiota can lead to a less hydrophobic bile acid pool, which benefits liver health, pinpointing the function microbiome of the gut-liver axis in immune and functional ageing [97]. Furthermore, the translocation of Enterobacteriaceae has been observed to reverse premature ageing in SIRT6 knockout mice, suggesting the potential of microbiome replacement or modulation in anti-ageing strategies [98].

Recently, the alternations of extracellular matrix (ECM) have emerged as a driver of ageing [99]. For example, the remodelling of collagen, the most abundant protein in ECM, is required for longevity in *C.elegans*, suggesting that ECM homeostasis represents a novel mechanism for healthy ageing [100]. Aging is associated with defects in cell turnover and tissue renewal, and reductions in stem cell activity and number, all of which alter with age. These processes are intricately modulated by the “niche” or the local extracellular matrix (ECM) surrounding the cells [101].

Recent research reveals that diapause, a natural phenomenon that happens in many species, including *C.elegans*, certain insects, and killifish, can be a powerful model to investigate stem cell, rejuvenation and ageing, underscoring the interconnections between different hallmarks of ageing [102].

### Other emerging technologies for gerotherapeutics

In the evolving landscape of gerotherapeutics, diverse findings and emerging technologies are reshaping the future of ageing interventions. Of note, the G-alpha protein, G_αq_ EGL-30, plays a crucial role in enhancing memory and overall healthspan in a conserved manner from *C.elegans* to mice, implying the potential for unexpected targeted therapies aimed at preserving cognitive function in ageing populations [103]. Similarly, newly developed enzyme-based therapies are targeting age-related macular degeneration, providing hope for individuals at risk of vision loss due to ageing [104]. Cryopreservation is advancing as a method for preserving cells and tissues for regenerative medicine applications, yet it remains under development [105]. Furthermore, novel techniques such as AI, microfluidic systems, and robotic-assisted high-throughput screening methods enable unbiased drug screening and direct measurement of lifespan and healthspan parameters in whole animals, significantly accelerating the study and testing of interventions for ageing [106–108].

## Healthy ageing and longevity medicine

### Lifestyle interventions, supplements and drugs for healthy ageing

In the evolving landscape of healthy ageing, clinical interventions rooted in exercise, diet, and pharmaceuticals are pivotal [61]. Accumulating evidence suggests that exercise operates beyond mere fitness and acts as a biological modifier by increasing circulating IL-6 levels to regulate inflammation, glucose homeostasis and lipid metabolism [109, 110], showcasing that exercise is not just a physical enhancer but also a potential biochemical modifier beneficial in countering the effects of ageing. Dietary approaches, such as calorie restriction, intermittent fasting, and fasting-mimicking diets (FMDs), have been shown to offer profound health benefits. In clinical settings, these diet strategies have led to notable improvements in patients with chronic conditions such as diabetes and hypertension [63, 111].

The integration of pharmaceuticals further broadens the anti-ageing intervention spectrum. Studies on natural compounds such as NAD+ precursors, alpha-ketobutyrate and urolithin-A showed improvements in mitochondrial function morphology [61, 72, 112]. The repurposing of drugs like rapamycin and other mTOR inhibitors, along with natural compounds like spermidine, can enhance autophagy and improve immune function and vaccine responses [113, 114], suggesting their potential to prevent age-related declines in the immune system. Similarly, combinations such as dasatinib and quercetin show improvements in reduced senescence marks and immune function among aged populations [83], highlighting the potential of pharmacological agents in extending healthspan.

These detailed insights into the multifaceted approaches encompassing exercise, diet, and pharmaceutical interventions reflect the comprehensive nature of interventions targeting ageing. They represent a holistic view that healthspan extension is achievable through targeted, evidence-based strategies grounded in rigorous clinical research and personalised healthcare paradigms.

## Transition from sick care to health-oriented longevity medicine

**Figure 2:**
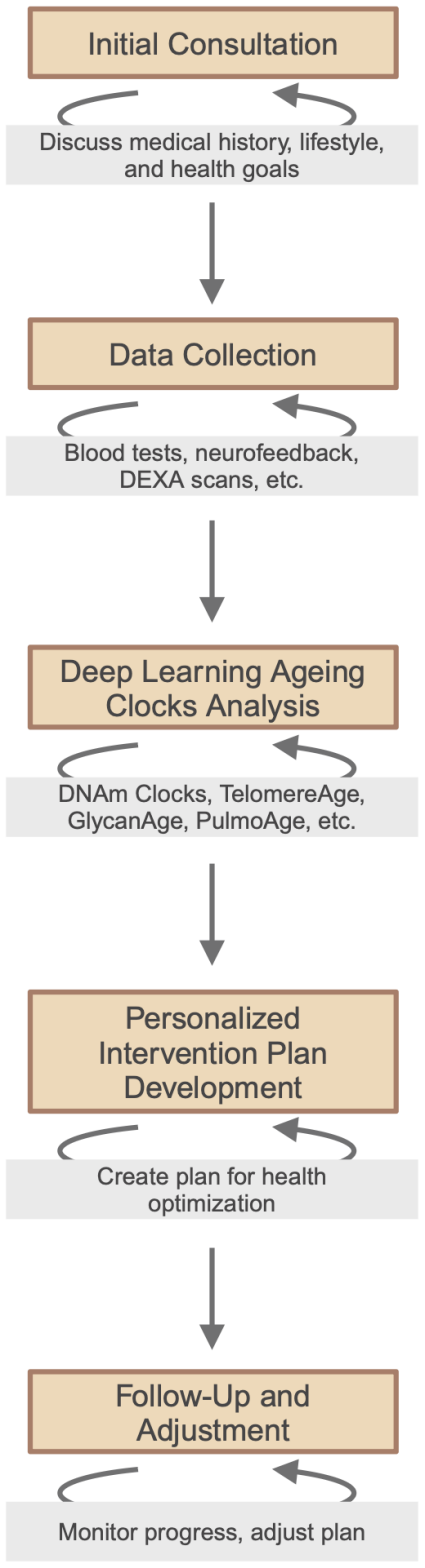
Framework of personalized, data-driven longevity medicine.

The emergence of longevity medicine, a personalised preventive medicine powered by deep biomarkers of ageing and longevity, is a paradigm shift from traditional, sickness-oriented healthcare towards a more proactive, AI-driven approach [115]. Highlighting the individualisation of patient care, this approach integrates data collection and analysis, from cellular biomarkers to advanced diagnostic techniques, to pave the road for more effective and personalised interventions [116]. Clinics specialising in longevity medicine are now adopting this data-driven approach, establishing longevity medicine protocols and standardized criteria, as well as laying the groundwork for broader accessibility and implementation of longevity interventions in clinical settings worldwide (Figure 2). New commercial platforms leverage the integration of blood biomarkers, DNA, physiological markers, health care records and user-generated data to optimize personal health and healthy longevity strategies [117].

The interrelation between longevity and cancer research is considerable, anchored in the shared hallmarks and biological processes between ageing and tumorigenesis [118]. Applying geroscience approaches to cancer and ageing can improve decision-making in oncology, particularly for patients who are not typically included in clinical trials due to age [116]. The cross-section between treatments for chronic diseases and ageing research sets the stage for dual-purpose drug discovery [119, 120], meaning to find drugs that not only treat individual age-related diseases but also target one or multiple hallmarks of ageing, addressing pathological aspects of ageing. For instance, the risk of cancer increases approximately 40 times from the age of 20 to the age of 60, showing that age is the major risk driver, but prevention and early detection can significantly lower its abundance in society before dual-purpose drugs become abundant. To achieve that, the transition from sickness-oriented to health-oriented medicine is crucial and will require the inclusion of healthy longevity medicine in medical education and advanced training for physicians, enriched by the current scientific advancements in ageing research.

## Ongoing challenges and future direction

Since 2019, the number of individuals over the age of 65 has surpassed those under 5, marking a significant demographic shift towards an ageing population (https://ourworldindata.org/age-structure). The majority of the elderly will have more than chronic disease associated with ageing, illustrating that curing a single age-related disease, while valuable, will not substantially change the health status of our global population [121, 122]. This transformation underscores the need for a comprehensive approach to extend healthspan and maintain workforce participation in older adults (https://www.un.org/en/global-issues/ageing). The urgency for systemic reforms spanning healthcare, policies, and societal norms is critical to alleviating the burdens posed by an ageing society. Advanced understanding of genetic, epigenetic, and environmental factors becomes increasingly vital to develop targeted health interventions for people across their life course especially in individuals over 50. There is a growing recognition of the importance of factoring in wider environmental, behavioral, and social determinants of health, encapsulated by the ‘exposome’, that have profound effects on the human health trajectory and overall resilience as people age [123].

The economic ramifications highlight ageing as a profound societal challenge, emphasizing that targeting fundamental ageing process is predicted to yield significantly greater economic benefits compared to focusing solely on individual chronic diseases [124]. Consequently, the healthy longevity research and development sector is urged to evolve swiftly by embracing innovative approaches, such as gene and cell therapies, organ replacement, engineered cells, full cell simulation, and reversible cryostasis. Moreover, the development of new tools to measure biology in ever greater detail and precision, such as single-cell proteomics, metabolomics, non-invasive blood chemistry monitoring and more, is essential for advancing our understanding and interventions in ageing. Overcoming challenges related to capital accessibility, quality control, and regulatory barriers is essential for progress. Furthermore, fostering community engagement and advocacy, and leveraging decentralized science and blockchain technology could spur research and innovation, paving the way for a new era of funding and knowledge exchange within the scientific community [125, 126].

**Figure 3:**
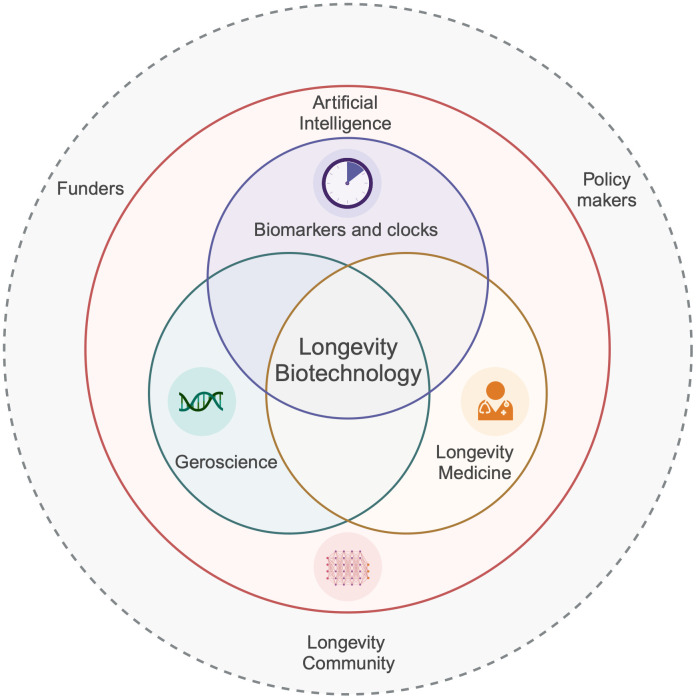
Integration of AI, biomarkers and clocks, geroscience, and longevity medicine in advancing human healthspan.

The integration of artificial intelligence (AI), biomarkers, ageing biology, and longevity medicine stands as a cornerstone for extending human healthy lifespan. AI innovations offer deeper insights and enable personalised strategies in drug discovery and clinical trials, bridging technology and biology. The integration of traditional biomarkers with advanced deep clocks is steering the shift towards personalised medical interventions. New ‘omics’ technologies coupled with novel AI analyses now enable the study of extended longevity mechanisms evolved in non-canonical model ageing extremists such as bats, whales and naked mole rats, uncovering which pathways are most relevant to humans. Collaboration across disciplines and borders, involving clinicians, biologists, data scientists, funders, policymakers and healthy longevity community, is crucial for the effective integration of scientific findings into practice (Figure 3). This collective approach not only aims to deepen our comprehension of ageing but also to forge new paths for enhancing healthspan, thereby improving the quality of life in later years and reducing the financial strains of age-related conditions.
